# Differences in perceived travel distance from central versus peripheral optic flow are the same when standing and walking

**DOI:** 10.1371/journal.pone.0348803

**Published:** 2026-05-08

**Authors:** Ambika Bansal, Hongyi Guo, Robert S. Allison, Laurence R. Harris

**Affiliations:** Centre for Vision Research, York University, Canada; University of Rochester, UNITED STATES OF AMERICA

## Abstract

The continuously evolving ways in which people move challenges our ability to process self-motion. Previous research from our lab has shown that when seated, optic flow presented in the far periphery results in people feeling they moved further than when the same motion was presented over the full field or in the central field only. The literature is mixed on the relative weightings of visual and non-visual cues when estimating travel distance, and it is unknown how non-visual cues might affect the use of optic flow in the far periphery. Here, we used a large-field edgeless display to visually “move” participants. Participants were either (i) physically stationary (visual-only condition), (ii) performing a blindfolded walking task on a treadmill (blindfolded walking condition), or (iii) visually “moving” while walking on a treadmill (visual-and-treadmill condition). Optic flow simulating forward self-motion was presented either full field, in the central field (inside 40°), or in the far periphery (outside 180°). Participants estimated travel distances by stopping at the location of a previously seen target (Move-To-Target Task) or adjusting a target to indicate the distance of a previous movement (Adjust-Target Task). In the Move-To-Target task, peripheral optic flow led to higher gains (perceived travel distance/ actual travel distance) compared to the central field and full-field conditions during both the visual-only and visual-and-treadmill conditions. In the same task, the blindfolded walking condition also led to higher gains than the visual-only or visual-and-treadmill conditions. In the Adjust-Target task, there were no significant differences between conditions. There were also no interaction effects in either task between field-of-view, and whether participants were standing or walking. This implies that the high sensitivity to optic flow in the far periphery is a general feature of perceptual odometry even when integrating non-visual cues with visual cues.

## Introduction

Having an accurate perception of travel distance is essential for navigating and moving through the world. During natural self-motion, information from our sensory systems (visual, vestibular, proprioceptive, auditory) is integrated to create a coherent perceptual experience of self-motion [1]. It is known that this redundant sensory information can be combined in a statistically optimal way based on their relative reliability [2]. In more modern forms of self-motion (e.g., driving a car, experiencing simulated self-motion in virtual reality, etc.), these sensory inputs can be conflicting. As the scenarios in which we experience modern forms of self-motion increase, so has the interest in understanding the sensory processes involved in estimating travel distance. In this study, we investigate the perception of travel distance using visual and non-visual self-motion cues. Within the visual self-motion cues, we were specifically interested in how peripheral and central optic flow may differentially affect perceived travel distance.

Self-motion perception has primarily been studied under isolated individual sensory cue conditions. Investigations have typically used either visual self-motion cues or body-based cues alone. Past research has found that these reduced cue conditions result in similar travel distance estimations to those resulting when the same cue conditions are combined [3]. They showed that information can be extracted from one modality and responded to accurately in either the same or different sensory condition. A common test used to examine proprioceptive and vestibular contributions to perceived travel distance is the blindfolded walking task, in which participants walk either freely or on a treadmill without visual information. Using this task, it has been established that humans are able to accurately reproduce the distance to a previously seen target with blindfolded walking [4–7]. It has been suggested that the mechanism responsible for the accuracy in the blindfolded walking task is “step integration” (step length times step frequency times time) [8]. Evidence for using step integration as an odometric cue has also been seen in other animals, such as the desert ant [9–11].

In active movement scenarios, vestibular and proprioceptive inputs are often coupled. Some researchers have passively moved participants to uncouple these body-based cues and combined this with testing either with visual cues or in the dark to isolate the vestibular and visual contributions to the perception of travel distance [12]. Participants experienced constant acceleration self-motion either visually (using a virtual reality display), physically (moving passively in the dark), or by a combination of both visual and passive physical motion. Perceived travel distance when receiving a combination of both visual and physical motion was more similar to experiencing only physical motion, compared to when experiencing only visual motion. These findings highlight the explicit importance of vestibular inputs in estimating travel distance.

It seems that the relative contributions of the different sensory systems to self-motion perception may be both task and stimulus dependent. During rotational movements, proprioceptive information generates the most consistent and accurate self-motion perception, followed by vestibular and then visual cues [13]. For translational motion, one study had participants compare travel distance when visual information provided through a head-mounted display was either congruent or incongruent with proprioceptive information generated from cycling on a stationary bike [14]. They found that when visual and proprioceptive information was inconsistent, participants responded as if optic flow were the dominant source of information, though the presence of proprioceptive information improved the visually specified distance estimates even when the cues were incongruent. An earlier study had participants walking on a treadmill at a constant speed, while manipulating the magnitude of optic flow [15]. Their results also showed participants changed their movements to align with the optic flow manipulations more so than with the constant speed of the treadmill, although they did not completely rely on optic flow. These studies provide evidence for a higher weighting of visual cues over non-visual cues during linear self-motion perception, in contrast to the proprioceptive dominance in rotational self-motion. More recent research integrating visual cues with either walking on a treadmill [16] or free walking [17,18] found that, as in the earlier experiments on rotational movements, non-visual cues were weighted higher than visual when estimating travel distance. Clearly the relative sensory contributions to the perception of linear travel distance is still an open question.

Neurophysiology research has identified specific regions of the limbic cortex that are particularly specialized to process high-speed retinal motion in the far periphery [19,20]. These results suggest that fast retinal motion detected in the far periphery may be processed differently compared to motion in the rest of the visual field. Previous research from our lab has shown that optic flow presented only in the far periphery (beyond 180°) resulted in people feeling they had moved further than when the same motion was presented full field or in only the central field [21]. For those experiments, however, participants were seated and received only visual stimuli about their movement. Others have investigated the relative contributions of radial and laminar optic flow in the perception of travel distance, again while stationary [22]. They found that laminar flow (corresponding to similar pattern of optic flow in the peripheral field) led to subjects feeling like they had moved further compared to radial flow (which correspond to optic flow presented in the central field when moving forward). Although these studies were investigating perceived travel distance, they align with earlier research that suggests that peripheral vision may be more effective than central vision in evoking self-motion for forwards linear movement [23] and left-right linear movement [24], sway [25,26], a stronger sense of presence and cybersickness [27], and the perception of a faster travel speed for optic flow beyond the central ±30° [28]. Peripheral vision has also been shown to be vital in maintaining static balance [29,30] and establishing spatial structure for navigation [31]. These studies together provide evidence for the importance of the periphery in optic flow processing, though few studies have looked at optic flow beyond the central ±90°. In the present study, we were not only interested in how the location of visual information (full field, central ±20°, far periphery beyond the central ±90°) would modulate the weightings of the visual contributions to perceived travel distance, but we were also interested in how the integration of visual and non-visual cues might affect the use of peripheral optic flow in self-motion perception. If non-visual cues are weighted higher than visual cues when estimating travel distance [16–18], the effects of visual field exposure, specifically in the far periphery, might diminish when visual and non-visual cues are combined.

There are two tasks that have historically been used to test the perception of travel distance. The first is the Move-To-Target task, in which participants judge travel distances by stopping at the location of a previously seen target. The second is the Adjust-Target task, in which participants adjust a target to indicate the extent of a previous movement. In the Move-To-Target task people tend to make underestimations in which they stop before the previously seen target location, and in the Adjust-Target task people tend to make overestimations in which they adjust the target further away than the actual extent of their previous travel [32,33]. We investigated the estimation of travel distance when optic flow was presented in the peripheral field compared to the central field, while participants were either walking or stationary. We predicted that, similar to previous research [21], participants would feel that they had moved further when receiving only peripheral optic flow, compared to when receiving optic flow in the full field or in the central field only. We also hypothesized that if non-visual cues were weighted higher than visual cues when estimating travel distance, the peripheral enhancement would diminish when visual and non-visual cues were combined.

## Methods

### Participants

We collected data from 18 participants (8M, 10F; mean age 20.3 yrs, SD ± 2.2). The recruitment period was between April 11^th^, 2024 and September 23^rd^, 2024. Participants were recruited using York’s Kinesiology Undergraduate Research Participant Pool and given course credit. All participants had normal or corrected-to-normal vision. By self-report, all participants had normal proprioceptive and vestibular function. Although, no clinical assessment methods were administered. All participants gave prior informed written consent and were naive to the purpose of the study. The protocols used in this study were approved by the York Human Participants Review Sub-committee (#e2024-024) and were conducted in accordance with the principles of the Declaration of Helsinki.

### Equipment

**Fig 1:**
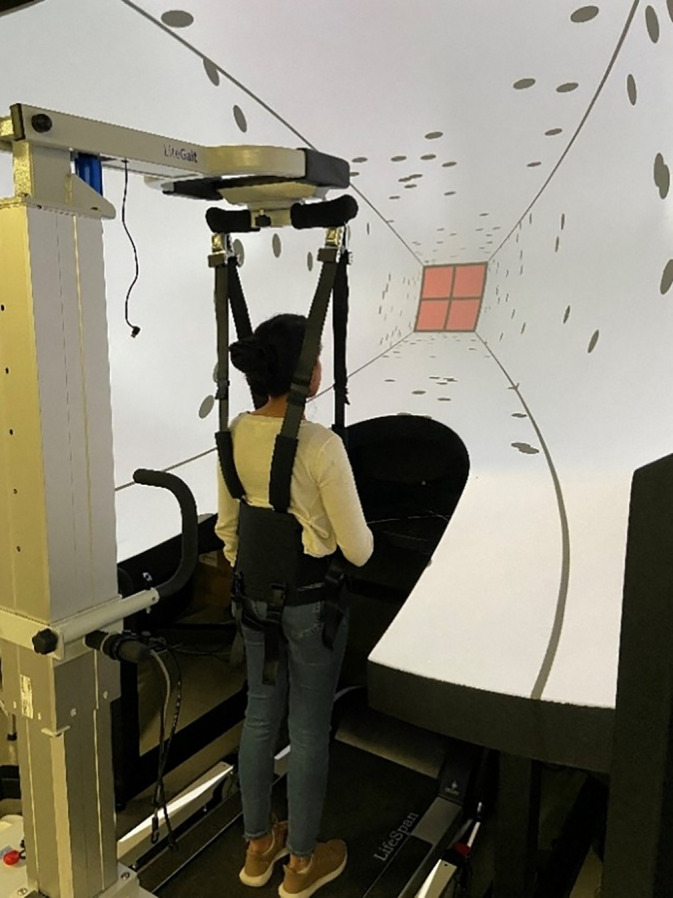
Experimental Setup. Participant stood or walked on a treadmill in front of the large-field Edgeless Graphics Geometry display, while strapped into a safety harness. Room lighting was extinguished during the experiments.

Visual stimuli were presented on a large-field Edgeless Graphics Geometry display (EGG, Christie, Canada, field of view ±112° horizontally). Participants were strapped into a safety harness (LG 300, LiteGait Training) and walked or stood on a LifeSpan TR5000-DT5 treadmill (see Fig 1). Both the display and the treadmill received input from the same computer that was used to generate stimuli. Responses were made using a standard Xbox controller.

### Stimuli

**Fig 2:**
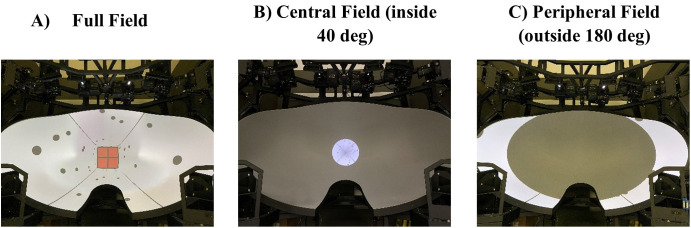
Visual stimuli. A) On the left is the simulated corridor and target square for the full field display (±112°). The target square was only present for distance responses (Adjust-Target Task) or before the trial (Move-To-Target Task) and was not present during the optic flow stimulus B) In the centre is the central field display (central inside 40°). C) On the right is the peripheral field display (outside 180°).

Participants experienced optic flow and/or physically walked on the treadmill at 1 m/s. They began either task while immersed in a simulated horizontal corridor (3.04 m tall x 3.04 m wide x 70 m long) displayed on the Edgeless Graphics Geometry screen. The walls of the corridor were white outlined with black edges and textured with 240 randomly placed black dots (radius 0.2 m to provide optic flow information. The black dots disappeared and reappeared at random intervals (0–6 s) and locations within 30 m from the observer’s position such that they could not be used as landmarks. In both tasks, the target was a red square (3.04 m x 3.04 m) with a black cross (line width 0.14 m) on it that filled the full corridor (Fig 2A). This target square was removed during the optic flow stimulus and only present during distance adjustment (Adjust-Target Task) after the trial or target distance presentation (Move-To-Target Task) before the trial.

During the motion stimulus, participants received optic flow information over either the full field of view where the whole screen was visible (Fig 2A), the central field where optic flow was presented only within inside 40° (Fig 2B), or the peripheral field where optic flow was only presented outside 180° (Fig 2C). These optic flow field-of-view (FOV) conditions were used during two sensory conditions: visual-only and visual-and-treadmill. There was also another sensory condition of blindfolded walking on the treadmill (no visual cues), resulting in a total of 7 conditions. The experiment was programmed in Python 3 with WorldViz Vizard VR toolbox (version 7.0).

### Move-To-Target Task

Each trial started with a simulated red square target presented at a pre-specified distance in the full-field corridor (Fig 2A). Participants pressed a button triggering the target to disappear and simulated motion towards the target’s position to commence. For the visual-and-treadmill condition, participants experienced optic flow on the screen while walking on the treadmill at the equivalent speed. For the visual-only condition, participants experienced optic flow on the screen while standing stationary on the treadmill. For the treadmill-only condition, participants first saw the target on the screen and then once the button was pressed, the visual display turned grey, and participants started walking on the treadmill. In all cases, participants indicated when they felt their nose had touched the previously seen target by pressing the button.

### Adjust-Target Task

Participants stood in the same corridor as for the Move-To-Target task. They first experienced simulated movement forward through a pre-specified distance, again created using optic flow and/or by walking on the treadmill. Once they had traveled through the target distance, the motion stopped, and the target appeared at a random position in front of them. Participants then used the joystick on the controller to slide the target back and forth along the corridor until it appeared as far away as the distance through which they felt they had just moved. They pressed a button to end the trial and move on to the next trial.

### Procedure

After providing informed consent, participants stood on the treadmill and were strapped into the safety harness that surrounded the treadmill, in front of the EGG display (Fig 1). After the instructions were explained, participants were given a practice session using the visual-and- treadmill condition, which included five trials at randomized distances between 5-32m. Once the practice was completed, participants ran the full version of each task.

This study was a within-subjects design such that every participant completed both the Move-To-Target and Adjust-Target tasks. The order in which the tasks were completed was counterbalanced, such that half completed the Move-To-Target first and the other half completed the Adjust-Target task first. Each task consisted of six target distances (5, 10, 15, 20, 25, 32 m), three field-of-view (FOV) conditions (full field, central field only, peripheral field only), and three sensory conditions (visual-only, visual-and-treadmill, treadmill-only). In all cases, the simulated self-motion speed and/or treadmill speed was 1 m/s. The sequence of distances presented was randomized to control for any order effects. The tasks were blocked by FOV condition and sensory condition, and the order in which these blocks were presented was randomized between participants. The whole experiment took about 1 hour (30 minutes for each of the two tasks).

### Data analysis

Each participant completed 84 trials ([3 FOV conditions x 2 sensory conditions + 1 treadmill only sensory condition] x 6 distances x 2 tasks). First, an outlier removal was completed. The outlier removal was performed at the group level for each distance in every condition (7 sensory conditions x 2 tasks). Any data less than the ‘Lower Quartile - 1.5 x Interquartile Range’ or above the ‘Upper Quartile + 1.5 x Interquartile Range’ were removed. Out of 1552 data points (84 trials x 18 participants), 70 data points were removed.

The raw gains were then calculated for each trial for both tasks. In both tasks, raw gains were calculated by dividing the perceived travel distance by the actual travel distance. In the Move-To-Target task, this translates to:


$${\text{Gain}}_{\text{Move}-\text{To}-\text{Target}}\;=\text{ Target Distance }/\text{ Self}-\text{Motion Distance}$$ (1)

For the Adjust-Target task, this translates to:


$${\text{Gain}}_{\text{Adjust}-\text{Target}}\;=\text{ Adjusted Target Distance }/\text{ Self}-\text{Motion Distance}$$ (2)

Perfect performance in both tasks would result in a raw gain of 1. In both tasks, a raw gain greater than 1 would imply that participants felt that they had moved further than the simulated distance. We started by first testing whether any effects of FOV and sensory conditions differed between tasks. Since we did find differences between tasks, we analyzed them separately and did not pool the data from both tasks. We also found a significant difference in variability between the tasks. A Linear Mixed Model (LMM) analysis was then performed on the raw gains for each task (collapsing across distances) using the lme4 [34] for R (version 4.3.0). We started with a base model where the fixed-effect structure was chosen as a function of our hypotheses, in which we were interested in the main effects of FOV and sensory condition. We compared this base model to a model that included an interaction term between FOV and sensory condition. The interaction model in this case chooses which interactions exist and ignores the rest. Since the treadmill only condition did not include the FOV conditions, this study was not a crossed factorial design, and therefore the treadmill only condition and FOV interaction would be ignored. Since no significant differences were found between the base model and interaction models for both the Move-To-Target (p = 0.37) and Adjust-Target (p = 0.22) tasks, this suggests that there were no interaction effects, and therefore, the interaction term was left out of the final model structure. Given that this study was not a crossed factorial design, we decided to test the main effects of sensory condition and FOV with two separate LMMs and compare the individual levels of each experimental variable against each other using the grand means of the other as intercept. For testing FOV we used the grand means of the sensory condition as intercept. For testing the sensory condition, we used the grand means of the FOV conditions as intercept. The final model structure for the LMM of the raw gains for FOV reads as follows:


Gain ~ FOV + Mean Sensory Condition + (FOV + Mean Sensory Condition | Participant) (3)

The final model structure for the LMM of the raw gains for Sensory Condition reads as follows:


Gain ~ Sensory Condition + Mean FOV + (Sensory Condition + Mean FOV | Participant) (4)

To test for statistical significance, we then computed bootstrapped confidence intervals at an alpha level of 0.05 using the confint() function from the base R with the “boot” argument and default settings otherwise. Using three separate ANOVAs, we also analyzed differences in variance between conditions for each Move-To-Target and Adjust-Target tasks, and between tasks. All data was analyzed in R (version 4.3.0). All data and data analysis can be found at (https://github.com/ambikabansal/FOV_Treadmill).

## Results

### Effect of Field-Of-View

### Move-To-Target Task

**Fig 3:**
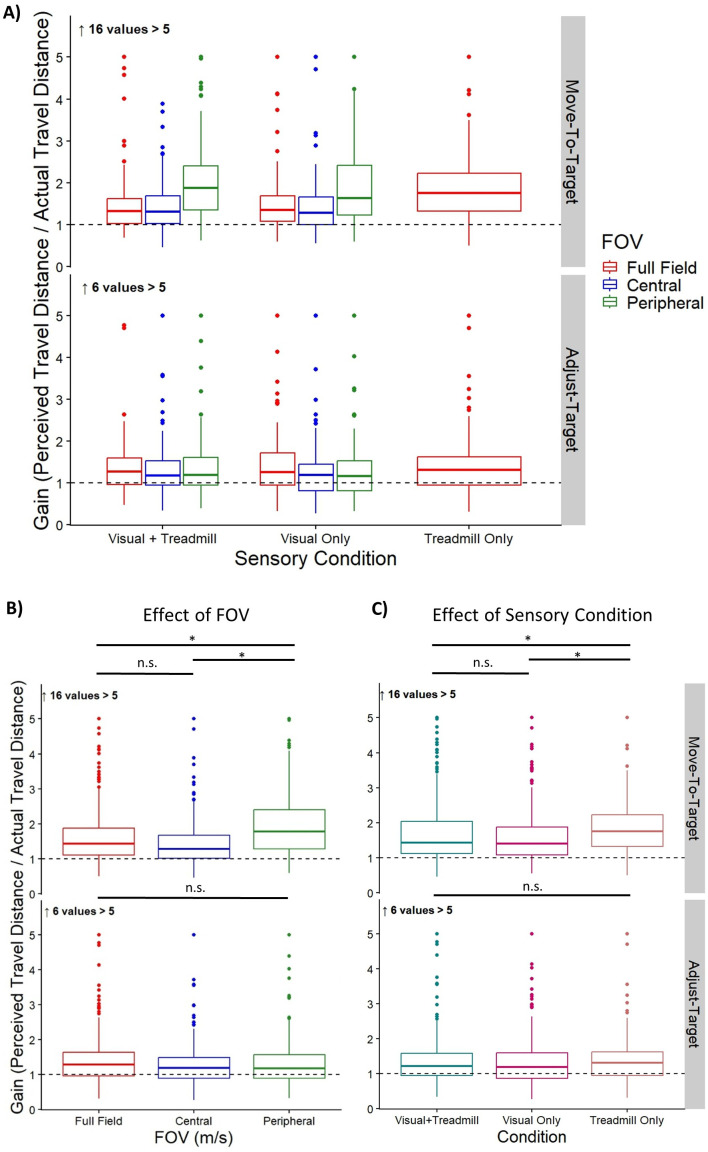
Raw Gains. (A) Box plots of the group raw gains for both the Move-To-Target (top row) and Adjust-Target (bottom row) tasks for each FOV and Sensory Condition. Data plotted here is from the full data set, with the y-axis restricted to gains below 5. The Move-To-Target task had 16 off-chart values. The Adjust-Target task had 6 off-chart values. The middle line represents the median, the boxes extend from the first quartile to the third quartile, the whiskers extend up to 1.5 times the interquartile range, and the outliers are shown as individual points beyond the whiskers. The full field is represented in red (left most), central field in blue (second from left), and peripheral field in green (right most). The black dashed line represents perfect performance (raw gain of 1). (B) Group raw gains collapsed across Sensory Condition to show the effect of FOV condition. (C) Group raw gains collapsed across FOV condition to show the effect of Sensory Condition. The Visual-and-Treadmill condition is represented in teal (left most), the Visual Only condition in pink (middle), and the Treadmill Only condition in tan (right most).

Table 1 shows the results from the linear mixed model for the effect of FOV on the raw gains of the Move-To-Target task. The peripheral condition resulted in significantly higher raw gains than the full-field, or central conditions (see Table 2). However, we found no significant differences between the full-field and central conditions. The raw gains from the Move-To-Target task across FOV conditions are shown at the top of Fig 3B.

**Table 1:** Results from the Linear Mixed Model run on data from the Move-To-Target task with the raw gain set as the dependent variable, with both FOV and Sensory Condition set as fixed effects. This table reports differences in FOV. This table reports unstandardized regression coefficients.

	Regression Coefficient	Standard Error	95% CI (lower)	95% CI (upper)	Significance
Full Field vs. Central	−0.07	0.09	−0.24	0.10	n.s.
Full Field vs. Peripheral	0.52	0.14	0.24	0.81	*
Central vs. Peripheral	−0.59	0.13	−0.85	−0.34	*

**Table 2:** Means and standard deviations of the raw gains for the different FOV conditions.

	Full Field	Central Field	Peripheral Field
Move-To-Target Task	1.67 ± 0.88	1.49 ± 0.75	2.12 ± 1.20
Adjust-Target Task	1.33 ± 0.61	1.23 ± 0.54	1.23 ± 0.54

### Adjust-Target Task

Table 3 shows the results from the linear mixed model for the effect of FOV on the raw gains of the Adjust-Target task. There were no significant differences between the full-field, central field, or peripheral conditions. The raw gains are from the Adjust-Target task across FOV conditions are shown at the bottom of Fig 3B.

**Table 3:** Results from the Linear Mixed Model run on data from the Adjust-Target task with the raw gain set as the dependent variable, with both FOV and Sensory Condition set as fixed effects. This table reports differences in the FOV. This table reports unstandardized regression coefficients.

	Regression Coefficient	Standard Error	95% CI (lower)	95% CI (upper)	Significance
Full Field vs. Central	−0.10	0.05	−0.21	0.002	n.s.
Full Field vs. Peripheral	−0.09	0.05	−0.19	0.0004	n.s.
Central vs. Peripheral	−0.003	0.05	−0.009	0.19	n.s.

### Effect of sensory condition

### Move-To-Target Task

Table 5 shows the results from the linear mixed model for the effect of sensory condition on the raw gains of the Move-To-Target task. The treadmill condition resulted in significantly higher raw gains than the visual-and-treadmill, or visual only conditions (see Table 4). However, we found no significant differences between the visual-and-treadmill and visual only conditions. The raw gains from the Move-To-Target task across sensory conditions are shown at the top of Fig 3C.

**Table 4:** Means and standard deviations of the raw gains for the different sensory conditions.

	Visual & Treadmill	Visual Only	Treadmill Only
Move-To-Target Task	1.69 ± 0.82	1.69 ± 1.05	2.05 ± 1.15
Adjust-Target Task	1.26 ± 0.49	1.26 ± 0.61	1.36 ± 0.67

**Table 5:** Results from the Linear Mixed Model run on data from the Move-To-Target task with the raw gain set as the dependent variable, with both FOV and Sensory Condition set as fixed effects. This table reports differences in Sensory Condition. This table reports unstandardized regression coefficients.

	Regression Coefficient	Standard Error	95% CI (lower)	95% CI (upper)	Significance
Visual & Treadmill vs. Visual Only	0.04	0.11	−0.17	0.24	n.s.
Visual & Treadmill vs. Treadmill Only	0.62	0.19	0.20	0.98	*
Visual Only vs. Treadmill Only	−0.57	0.22	−0.99	−0.16	*

### Adjust-Target Task

Table 6 shows the results from the linear mixed model for the effect of sensory condition on the raw gains of the Adjust-Target task. There were no significant differences between the visual-and-treadmill, visual only, or treadmill only conditions. The raw gains from the Adjust-Target task across sensory conditions are shown at the bottom of Fig 3C.

**Table 6:** Results from the Linear Mixed Model run on data from the Adjust-Target task with the raw gain set as the dependent variable, with both FOV and Sensory Condition set as fixed effects. This table reports differences in Sensory Condition. This table reports unstandardized regression coefficients.

	Regression Coefficient	Standard Error	95% CI (lower)	95% CI (upper)	Significance
Visual & Treadmill vs. Visual Only	−0.0008	0.07	−0.14	0.14	n.s.
Visual & Treadmill vs. Treadmill Only	0.05	0.08	−0.10	0.21	n.s.
Visual Only vs. Treadmill Only	−0.05	0.11	−0.26	0.17	n.s.

**Fig 4:**
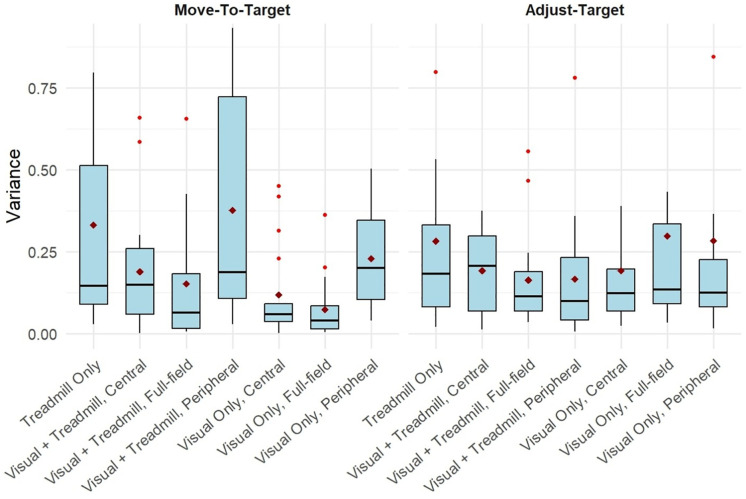
Variance. Box plots of the group variance for both the Move-To-Target (left) and Adjust-Target (right) tasks for each FOV and Sensory Condition. The middle line represents the median, the boxes extend from the first quartile to the third quartile, the whiskers extend up to 1.5 times the interquartile range, and the outliers are shown as individual points beyond the whiskers. The diamond points represent the mean.

There are no significant differences in variance between conditions in the Move-To-Target (p = 0.23) or Adjust-Target (p = 0.12) tasks, or between tasks (p = 0.58) (Fig 4).

## Discussion

This study investigated how the presence of both visual and non-visual cues affected the estimation of travel distance when optic flow was presented in the far peripheral field (outside 180°) compared to when it was presented over the full-field or in just the central field (inside 40°). We found that optic flow presented in the periphery led to people feeling they had moved further than when optic flow was presented to either the central field or the full field in the Move-To-Target task. For the same Move-To-Target task, we found that blindfolded treadmill walking led to people feeling they had moved further than when they were treadmill walking with optic flow information or receiving optic flow information while stationary. However, we found no significant differences between any of our conditions with the Adjust-Target task. We also found no interaction effect between which part of the visual field was presenting the optic flow and whether participants were walking or not.

### Effect of FOV condition

Clearly, our findings extend the results of previous research. Similar to previous research using seated observers, our participants responded earlier on the Move-to-Target task in the peripheral FOV condition compared to the full field or central field [21]. This was true for both the visual only and visual-and-treadmill conditions. However, we found no significant differences between these conditions in the responses to the Adjust-Target task (not tested by [21]). Our findings also align with previous research from our lab that investigated perceived travel distance using laminar and radial optic flow [22]. That study found that laminar flow (found only in the peripheral field when moving forwards) led to higher raw gains compared to radial flow (corresponding to optic flow presented in the central field when moving forward). These higher gains found when only peripheral optic flow was available also supports previous research that suggests that peripheral vision may be more effective than central in evoking self-motion [23], and sway [25]. When experiencing forward self-motion, the angular velocities of objects in a scene are dependent on their position relative to the heading of the observer, their distance and on the speed of the moving observer [35]. Information in the peripheral visual field is associated with higher angular velocities, while information from the central visual field are associated with lower angular velocities. Similarly features on the walls in our stimulus had the smalled egocentric radial distance and thus increased velocity when they were in the coronal plane through the eyes (i.e., at 90º eccentricity). Previous research has found that perceived travel speed was higher when visual information was only present in the peripheral FOV compared to when it was available in the central FOV or over the full field [28,36]. This remained true when optic flow was provided with a less realistic random dot pattern [28] or with a more realistic driving simulator [36]. Across both studies, these authors hypothesized that during forward self-motion, the availability of lower angular velocities from the central FOV directly decreases the overall estimation of speed. In the present study, this would explain the lower raw gains in both the central FOV and full field conditions, compared to the peripheral-only FOV. The greater sensitivity to optic flow in the periphery also makes sense from an evolutionary standpoint, as movement seen only in the far periphery may signal a greater threat. This potential danger may have driven the evolution of an increase in our general motion sensitivity in the periphery, thereby enhancing our sensitivity to optic flow [37]. Overestimating movements in the far periphery could provide a survival advantage by promoting more caution.

Although the human field of view is close to ±110° (Strasburger et al., 2011), most research studies investigating the effects of central and peripheral optic flow have not been able to examine optic flow in the far periphery due to their limitations in display size. Here, we were able to examine the less researched far periphery using a large-field Edgeless Graphics Geometry display (field of view ±112°). Previous research using this same display, but in a non-stereoscopic mode, found that there was a significant difference between using the far periphery (beyond 90°) and the less far periphery (beyond 40°) when estimating travel distance, which is why we were specifically interested in examining optic flow in the far periphery [21]. This display however did not have stereoscopic depth cues, which would have introduced a conflict between the monocular and binocular depth cues in the central field. Although earlier studies have found stereoscopic cues to enhance vection [38–39], later research has shown that there is no effect on stereoscopic depth cues on estimating travel distance [40]. Although binocular cues would not have played a role in the far periphery which is only monocularly visible, this disparity between monocular and binocular depth cues in the central field could have also contributed to the differences we see between the FOV conditions.

### Effect of sensory condition

We found that for the Move-To-Target task, blindfolded treadmill walking led to higher raw gains than treadmill walking with visual information or when physically stationary viewing visual information alone. However, we found no significant differences between the visual-and- treadmill condition and the visual only condition. We also found no significant differences with the Adjust-Target task. In the treadmill only condition of the Move-To-Target task, where we find larger raw gains, the target distance was presented visually, and participants were required to transform that visual distance information into treadmill walking distance. In the visual only and visual-and-treadmill conditions, the visual motion information could be used to estimate the visual target distance. Previous research has shown that a visually presented target can be more accurately matched when making visual travel distance estimates, compared to when making estimates using a passive physical movement [20,41]. This is true for both short [41] and long travel distance estimates [20]. Our findings from the Adjust-Target task also align with previous research, who found no differences in travel distance estimates between isolated visual only and body-based only conditions and sensory combined conditions [3]. These findings are however contrary to previous research that has provided evidence that body-based cues can be weighted higher than visual cues when estimating travel distances using active physical movement, both when free walking [17,18] and walking on a treadmill [16]. The current study taken in combination with the previous literature suggests that the sensory contributions to the perception of travel distance might be task and stimulus dependent. Previously, our group has also investigated the visual and vestibular contributions to the perception of travel distance during long-term exposure to microgravity using the Move-To-Target task [42]. We found that there were no significant differences between the perception of travel distance on Earth compared to in space during long-term exposure to microgravity. Although, we did find small differences in perceived travel distance on Earth when completing the task in a supine position compared to a seated one. This study also provides mixed evidence as to the sensory weightings and sensory integration when perceiving travel distance. It should be noted that here, we had participants walking with their eyes open in front of a grey screen instead of walking with their eyes closed. The effects may change if there was no visual input.

## Conclusions

These findings support the idea that when estimating travel distance, non-visual cues may not be weighted higher than visual cues. These findings also highlight the importance of the far periphery in self-motion processing, and that these differences in perceived travel distance when using central versus peripheral optic flow are the same when walking and standing. However, the differences in perceived travel distance were only found in the Move-To-Target task and not in the Adjust-Target task, which implies that different computations may be used to estimate travel distance in these two Move-To-Target and Adjust-Target tasks.

## References

[1] Laurienti PJ, Burdette JH, Maldjian JA, Wallace MT. Enhanced multisensory integration in older adults. Neurobiol Aging. 2006;27(8):1155–63. doi: 10.1016/j.neurobiolaging.2005.05.024 16039016

[2] Ernst MO, Banks MS. Humans integrate visual and haptic information in a statistically optimal fashion. Nature. 2002;415(6870):429–33. doi: 10.1038/415429a 11807554

[3] Sun H-J, Campos JL, Young M, Chan GSW, Ellard CG. The contributions of static visual cues, nonvisual cues, and optic flow in distance estimation. Perception. 2004;33(1):49–65. doi: 10.1068/p5145 15035328

[4] Fukusima SS, Loomis JM, Da Silva JA. Visual perception of egocentric distance as assessed by triangulation. J Exp Psychol Hum Percept Perform. 1997;23(1):86–100. doi: 10.1037//0096-1523.23.1.86 9090148

[5] Loomis JM, Da Silva JA, Fujita N, Fukusima SS. Visual space perception and visually directed action. J Exp Psychol Hum Percept Perform. 1992;18(4):906–21. doi: 10.1037//0096-1523.18.4.906 1431754

[6] Mittelstaedt ML, Mittelstaedt H. Idiothetic navigation in humans: estimation of path length. Exp Brain Res. 2001;139(3):318–32. doi: 10.1007/s002210100735 11545471

[7] Rieser JJ, Ashmead DH, Talor CR, Youngquist GA. Visual perception and the guidance of locomotion without vision to previously seen targets. Perception. 1990;19(5):675–89. doi: 10.1068/p190675 2103000

[8] Durgin FH, Akagi M, Gallistel CR, Haiken W. The precision of locomotor odometry in humans. Exp Brain Res. 2009;193(3):429–36. doi: 10.1007/s00221-008-1640-1 19030852

[9] Thiélin-Bescond M, Beugnon G. Vision-independent odometry in the ant Cataglyphis cursor. Naturwissenschaften. 2005;92(4):193–7. doi: 10.1007/s00114-005-0609-1 15772808

[10] Wittlinger M, Wehner R, Wolf H. The ant odometer: Stepping on stilts and stumps. Science. 2006;312(5782):1965–7. doi: 10.1126/science.1126912 16809544

[11] Wittlinger M, Wehner R, Wolf H. The desert ant odometer: a stride integrator that accounts for stride length and walking speed. J Exp Biol. 2007;210(Pt 2):198–207. doi: 10.1242/jeb.02657 17210957

[12] Harris LR, Jenkin M, Zikovitz DC. Visual and non-visual cues in the perception of linear self-motion. Exp Brain Res. 2000;135(1):12–21. doi: 10.1007/s002210000504 11104123

[13] Bakker NH, Werkhoven PJ, Passenier PO. The effects of proprioceptive and visual feedback on geographical orientation in virtual environments. Presence: Teleoperators & Virtual Environments. 1999;8(1):36–53. doi: 10.1162/105474699566035

[14] Sun H-J, Campos JL, Chan GSW. Multisensory integration in the estimation of relative path length. Exp Brain Res. 2004;154(2):246–54. doi: 10.1007/s00221-003-1652-9 14685814

[15] Prokop T, Schubert M, Berger W. Visual influence on human locomotion modulation to changes in optic flow: Modulation to changes in optic flow. Experimental Brain Research. 1997;114(1):63–70. doi: 10.1007/PL00005624 9125452

[16] Kopiske K, Heinrich E-M, Jahn G, Bendixen A, Einhäuser W. Multisensory cues for walking in virtual reality: humans combine conflicting visual and self-motion information to reproduce distances. J Neurophysiol. 2023;130(4):1028–40. doi: 10.1152/jn.00011.2023 37701952

[17] Campos JL, Byrne P, Sun H-J. The brain weights body-based cues higher than vision when estimating walked distances. Eur J Neurosci. 2010;31(10):1889–98. doi: 10.1111/j.1460-9568.2010.07212.x 20584194

[18] Campos JL, Butler JS, Bülthoff HH. Multisensory integration in the estimation of walked distances. Exp Brain Res. 2012;218(4):551–65. doi: 10.1007/s00221-012-3048-1 22411581

[19] Rockland KS. Visual system: prostriata--a visual area off the beaten path. Curr Biol. 2012;22(14):R571-3. doi: 10.1016/j.cub.2012.05.030 22835792

[20] Yu H-H, Chaplin TA, Davies AJ, Verma R, Rosa MGP. A specialized area in limbic cortex for fast analysis of peripheral vision. Curr Biol. 2012;22(14):1351–7. doi: 10.1016/j.cub.2012.05.029 22704993

[21] McManus M, D’Amour S, Harris LR. Using optic flow in the far peripheral field. J Vis. 2017;17(8):3. doi: 10.1167/17.8.3 28672369

[22] Harris LR, Herpers R, Jenkin M, Allison RS, Jenkin H, Kapralos B, et al. The relative contributions of radial and laminar optic flow to the perception of linear self-motion. J Vis. 2012;12(10):7. doi: 10.1167/12.10.7 22976397

[23] Brandt T, Dichgans J, Koenig E. Differential effects of central verses peripheral vision on egocentric and exocentric motion perception. Exp Brain Res. 1973;16(5):476–91. doi: 10.1007/BF00234474 4695777

[24] Tarita-Nistor L, González EG, Spigelman AJ, Steinbach MJ. Linear vection as a function of stimulus eccentricity, visual angle, and fixation. J Vestib Res. 2006;16(6):265–72. doi: 10.3233/ves-2006-16603 17726279

[25] Delorme A, Martin C. Roles of retinal periphery and depth periphery in linear vection and visual control of standing in humans. Can J Psychol. 1986;40(2):176–87. doi: 10.1037/h0080091 3730954

[26] Kawakita T, Kuno S, Miyake Y, Watanabe S. Body sway induced by depth linear vection in reference to central and peripheral visual field. Jpn J Physiol. 2000;50(3):315–21. doi: 10.2170/jjphysiol.50.315 11016981

[27] Lin JJ-W, Duh HBL, Parker DE, Abi-Rached H, Furness TA. Effects of field of view on presence, enjoyment, memory, and simulator sickness in a virtual environment. Proceedings IEEE Virtual Reality. 2002;164–71. 10.1109/vr.2002.996519

[28] Pretto P, Ogier M, Bülthoff HH, Bresciani J-P. Influence of the size of the field of view on motion perception. Computers & Graphics. 2009;33(2):139–46. doi: 10.1016/j.cag.2009.01.003

[29] Amblard B, Carblanc A. Role of foveal and peripheral visual information in maintenance of postural equilibrium in man. Percept Mot Skills. 1980;51(3 Pt 1):903–12. doi: 10.2466/pms.1980.51.3.903 7208238

[30] Dickinson J, Leonard JA. The role of peripheral vision in static balancing. Ergonomics. 1967;10(4):421–9. doi: 10.1080/00140136708930889 6073117

[31] Turano KA, Yu D, Hao L, Hicks JC. Optic-flow and egocentric-direction strategies in walking: central vs peripheral visual field. Vision Res. 2005;45(25–26):3117–32. doi: 10.1016/j.visres.2005.06.017 16084556

[32] Lappe M, Jenkin M, Harris LR. Travel distance estimation from visual motion by leaky path integration. Exp Brain Res. 2007;180(1):35–48. doi: 10.1007/s00221-006-0835-6 17221221

[33] Bansal A, McManus M, Jörges B, Harris LR. Perceived travel distance depends on the speed and direction of self-motion. PLoS One. 2024;19(9):e0305661. doi: 10.1371/journal.pone.0305661 39321156 PMC11423974

[34] Bates D, Mächler M, Bolker B, Walker S. Fitting Linear Mixed-Effects Models Using lme4. J Stat Soft. 2015;67(1). doi: 10.18637/jss.v067.i01

[35] Gibson JJ. The perception of the visual world. Cambridge: Riverside Press. 1950.

[36] Pretto P, Chatziastros A. Changes in optic flow and scene contrast affect the driving speed. Europe Driving Simulation Conference. 2006;263–72.

[37] Strasburger H, Rentschler I, Jüttner M. Peripheral vision and pattern recognition: a review. J Vis. 2011;11(5):13. doi: 10.1167/11.5.13 22207654 PMC11073400

[38] Palmisano S. Perceiving self-motion in depth: the role of stereoscopic motion and changing-size cues. Percept Psychophys. 1996;58(8):1168–76. doi: 10.3758/bf03207550 8961828

[39] Palmisano S. Consistent stereoscopic information increases the perceived speed of vection in depth. Perception. 2002;31(4):463–80. doi: 10.1068/p3321 12018791

[40] Frenz H, Lappe M, Kolesnik M, Bührmann T. Estimation of travel distance from visual motion in virtual environments. ACM Trans Appl Percept. 2007;4(1):3. doi: 10.1145/1227134.1227137

[41] Israël I, Chapuis N, Glasauer S, Charade O, Berthoz A. Estimation of passive horizontal linear whole-body displacement in humans. J Neurophysiol. 1993;70(3):1270–3. doi: 10.1152/jn.1993.70.3.1270 8229174

[42] Jörges B, Bury N, McManus M, Bansal A, Allison RS, Jenkin M, et al. The effects of long-term exposure to microgravity and body orientation relative to gravity on perceived traveled distance. NPJ Microgravity. 2024;10(1):28. doi: 10.1038/s41526-024-00376-6 38480736 PMC10937641

